# Continuity of care is an important and distinct aspect of childbirth experience: findings of a survey evaluating experienced continuity of care, experienced quality of care and women’s perception of labor

**DOI:** 10.1186/s12884-017-1615-y

**Published:** 2018-01-08

**Authors:** Hilde Perdok, Corine J. Verhoeven, Jeroen van Dillen, Tjerk Jan Schuitmaker, Karla Hoogendoorn, Jolanda Colli, François G. Schellevis, Ank de Jonge

**Affiliations:** 1 Department of Midwifery Science, Midwifery Academy Amsterdam/Groningen (AVAG) and EMGO Institute for Health and Care Research, VU University Medical Center, Amsterdam, The Netherlands and at Catharina Hospital, Van der Boechorststraat 7, 1081 BT Amsterdam, The Netherlands; 2 Department of Midwifery Science, AVAG and EMGO Institute for Health and Care Research, VU University Medical Center, Amsterdam, The Netherlands and at Maxima Medical Center, Veldhoven, The Netherlands; 3 Department of Obstetrics and Gynaecology, Radboud University Medical Center Nijmegen, Nijmegen, The Netherlands; 4 Faculty of Earth & Life Sciences, Athena Institute, VU University Medical Center, Amsterdam, The Netherlands; 5 Gelre Verloskundig Centrum Apeldoorn, Apeldoorn, The Netherlands; 6 Midwifery practice Oestgeest, The Netherlands and Co-operation of Midwives Leiden area (Cooperatie LEO), Leiden, The Netherlands; 7 Department of General Practice & Elderly Care Medicine, EMGO Institute for Health and Care Research, VU University Medical Center, Amsterdam, The Netherlands and Netherlands Institute for Health Services Research, Utrecht, The Netherlands

**Keywords:** Patient perspective, Birth, Labor, Childbirth experience, Continuity of care, Quality of care, Perception of care

## Abstract

**Background:**

To compare experienced continuity of care among women who received midwife-led versus obstetrician-led care. Secondly, to compare experienced continuity of care with a. experienced quality of care during labor and b. perception of labor.

**Methods:**

We conducted a questionnaire survey in a region in the Netherlands in 2014 among 790 women after they gave birth. To measure experienced continuity of care, the Nijmegen Continuity Questionnaire was used. Quality of care during labor was measured with the Pregnancy and Childbirth Questionnaire, and to measure perception of labor we used the Childbirth Perception Scale.

**Results:**

Three hundred twenty five women consented to participate (41%). Of these, 187 women completed the relevant questions in the online questionnaire. 136 (73%) women were in midwife-led care at the onset of labor, 15 (8%) were in obstetrician-led care throughout pregnancy and 36 (19%) were referred to obstetrician-led care during pregnancy. Experienced personal and team continuity of care during pregnancy were higher for women in midwife-led care compared to those in obstetrician-led care at the onset of labor. Experienced continuity of care was moderately correlated with experienced quality of care although not significantly so in all subgroups. A weak negative correlation was found between experienced personal continuity of care by the midwife and perception of labor.

**Conclusion:**

This study suggests that experienced continuity of care depends on the care context and is significantly higher for women who are in midwife-led compared to obstetrician-led care during labor. It will be a challenge to maintain the high level of experienced continuity of care in an integrated maternity care system.

Experienced continuity of care seems to be a distinctive concept that should not be confused with experienced quality of care or perception of labor and should be considered as a complementary aspect of quality of care.

**Electronic supplementary material:**

The online version of this article (10.1186/s12884-017-1615-y) contains supplementary material, which is available to authorized users.

## Background

Continuous support during labor from the same maternity caregiver has been associated with a positive childbirth experience [1, 2]. This is referred to as “relational continuity” or personal continuity which supports trust and familiarity between care provider and the patient. Other dimensions of continuity of care are “information continuity” in which the care provider uses and exchanges information on past events to deliver care that is appropriate to the patient’s current circumstances and “management continuity” in which the care providers connect their care in a coherent way [3].

Some studies suggest that personal continuity of care is related to fewer interventions such as the need for pain relief [4] and to feeling safer during labor [5]. Moreover, discontinuity of care (e.g. in case of referral to another care provider) could lead to unsafe situations due to more handovers and therefore loss of information [6], as well as inconsistency in advice and information from multiple caregivers.

Continuity of care is only one of the aspects, which can be measured when evaluating childbirth experience. Besides this, quality of care [7], satisfaction with care [8, 9] and perception of labor [10] are measured in studies evaluating childbirth experiences.

To measure quality of care, satisfaction scores are often used. Factors such as feeling supported [8, 11] care setting [12] and involvement in decision-making [8]have proven to be important for women’s satisfaction with care. However, satisfaction scores as a measure of patient perceived quality have limitations because patients do not easily express dissatisfaction [13] and they are strongly colored by expectations and prior experiences [14]. Tools for measuring satisfaction with maternity care have not been rigorously tested [15]. Nonetheless, literature suggests that women who were referred (during labor and birth) from midwife- to obstetrician-led care, and are cared for by multiple care providers, experience less satisfaction with their care [4]or less quality of care [1] compared to women who have not been referred. This could indicate that more personal continuity of care is associated with satisfaction and experienced quality of care.

A positive perception of labor is of importance as psychological distress during labor can contribute to the development of postnatal stress [16]or posttraumatic stress disorder [17]. It is not clear whether personal continuity of care is associated with perception of labor.

In the Netherlands, women at low risk for complications start their antenatal care with a primary care midwife in a midwife-led care context. Women who develop a risk factor or a complication during pregnancy or labor, as listed in the national “List of Obstetric indications” [18], are referred to secondary, obstetrician-led care. The number of referrals from midwife-led care to obstetrician-led care during pregnancy is approximately 35% and additionally, approximately 22% of women are referred during labor assets.perined.nl. Most of the latter women are referred for a “moderate risk” indication such as the need for pain medication, failure to progress during the first stage of labor or meconium stained liquor [19]. Referral often means that new care providers (clinical midwives, obstetricians) take over care for these women, which leads to discontinuity of personal care as the primary care midwife is no longer involved in the care.

Currently, the Netherlands is in a transition regarding the organisation of maternity care moving from separate midwife- and obstetrician-led care towards a system of integrated care; care will be delivered by professionals from multiple disciplines and across care setting boundaries in close collaboration.

On one hand, integrated care could improve personal continuity of care if women have one case manager regardless of their level of risk [20]. On the other hand, personal continuity of care may be reduced as more professionals are routinely involved in the care process, Therefore, it is important to evaluate the effect of integrated care on continuity, quality of care and perception of labor from women’s perspective.

To date, most studies limit their focus to either experienced continuity of care, experienced quality of care, satisfaction of care, or the perception of labor. We wanted to examine experienced continuity in relation to the level of care, and the associations between experienced continuity of care and other measures of childbirth experiences.

Therefore the aims of this study were:

- To compare experienced continuity of care during pregnancy and labor among women who were in midwife led versus obstetrician led care.
- To study the associations between experienced continuity of care and a. experienced quality of care during labor and b. perception of labor.

## Methods

### Study design

We conducted a survey among women having given birth in Leiden in the Netherlands in October 2014.

### Participants

Women were eligible if they answered the relevant questions and care during their puerperium was provided by one of the 10 primary care midwifery practices in the region of Leiden in the Netherlands in the period May till September 2014. Women with a primary caesarean section and women who gave birth to a child with a congenital abnormality were excluded.

### Procedure

During home visits in the puerperium, primary care midwives asked all women for written consent to participate in this study. These women either had midwife-led care, obstetrician-led care or received care from both the primary care midwife and hospital staff during pregnancy or birth in case of referral. Usually, primary care midwives take care of postnatal care of all women after childbirth, irrespective of the place of birth. A link to the online questionnaire, using Survayzer Nederland BV, was sent by e-mail to those women who gave written consent. The period between giving birth and sending the questionnaire was no longer than 6 months. Non-responders received a reminder by e-mail after 2 weeks. Only non-identifiable information was available for the researchers who analyzed the data.

The study was submitted to the medical ethics committee of VU University Medical Center (reference number 2014/030). An ethical approval was not considered necessary according to the Dutch legislation https://www.vumc.nl/afdelingen/METc/niet-wmo/beoordeling/.

### Measurements

Women’s experiences with care were measured with the Nijmegen Continuity Questionnaire (NCQ) [21], the validated Pregnancy and Childbirth Questionnaire (PCQ) [22] and the validated Childbirth Perception Scale (CPS) [23].

The NCQ [21] was used to assess experienced continuity of care. Originally, the NCQ questionnaire was developed for patients in general practice with a chronic disease, and the questionnaire has been adapted to maternity care. The NCQ is divided in three subscales measuring patients’ experienced personal continuity/the care provider knows me (subscale one), experienced personal continuity/ the care provider shows commitment (subscale two), team continuity within the same care setting and cross-boundary continuity of care between care settings (subscale three). The NCQ consists of 28 items, which are scored on a five-point Likert scale ranging from totally disagree to totally agree. A higher score indicates higher experienced continuity. The scores of the three subscales were calculated separately.

The PCQ [22]was used to assess the quality of obstetric care during labor as perceived by women. The PCQ is a 25-item scale, primarily based on the experiences and perceptions of pregnant women (18 items) and women who recently gave birth (7 items). In the PCQ questions are formulated in positive and negative statements, rated on a five point Likert scale, from totally agree (1) to totally disagree (5). For this research only the seven items regarding labor were used. The total range of this subscale is 35 points and after recoding, higher scores indicate a higher quality of care during labor.

The CPS [23] was used to assess the perception of labor. The CPS is a 12-item scale divided in two subscales of six items representing perception of labor and perception of the first week postpartum. For this research only the subscale related to labor was used. Each item is a statement to be scored on a four-point Likert scale ranging from totally agree (0) to totally disagree (3). The total range of this subscale is 18 points in which higher scores indicate a less positive perception of labor. For this study the scores were reversed resulting in higher scores indicating a more positive perception of labor.

### Statistical analyses

The data were analyzed using SPSS version 22.0 [24].

The subscale scores were calculated as the mean of the item scores in each subscale. The subscale score was only calculated if all items were applicable for the subgroup of women. Participants with more than one missing value within a subscale were excluded. Differences between means of subscale scores were tested with the t-test. A *P*-value of ≤0.05 was considered statistically significant.

Spearman correlation analyses were used to assess the correlation between continuity of care and experienced quality of care during labor and perception of labor. Coefficients between 0 and 0.30 were defined as a weak correlation, from 0.30 to 0.50 as moderate, and 0.50 or higher, as a strong correlation. Multivariable linear regression analyses were performed to adjust for parity, which might be associated with the experienced continuity, quality of care or perception of care. For women who were referred during pregnancy, both the scores for the primary care midwife and hospital staff were calculated. Women in midwife-led care during pregnancy and at the onset of labor were taken as the reference group and were compared with the other groups.

The analyses were not corrected for mode of birth because of low numbers.

## Results

Table 1 shows the women’s personal and obstetric characteristics.

**Table 1:** Patients’ characteristics and their obstetric features compared with regional data (all women who gave birth in the region in 2014) and national data including all births in the Netherlands in 2014

Characteristics	Respondents *n* = 187 n (%)	Regional data 2014 (*n* = 3085) %	National data PRN 2014 (*n* = 175,215) %
*Demographics*			
Age in years			
Mean [SD]	31.5 [4.1]	31	31 [4.9]
Educational level		–	–
Low	15 (8.0)		
Middle	52 (27.8)		
High	120 (64.2)		
Ethnicity			
Dutch	184 (98.4)	85	74
Other	3 (1.6)	15	26
*Obstetric features*			
Parity			
Primiparous	78 (41.7)	44	45
Parous	109 (58.3)	56	55
Gestational age during birth in weeks median [range]	40 [35–42]	39	40
Mode of birth			
Vaginal spontaneous	163(87.2)	72	75
Vaginal assisted (vacuum/forceps)	17 (9.1)	10	9
Caesarean section, not primary	7 (3.7)	18	17
Location of birth			
Home	54 (28.9)	12	15
Midwife-led hospital	45 (24.1)	13	13
Obstetrician-led hospital	88 (47.0)	75	71
Care during pregnancy			
Midwife-led care	136 (72.7)	79^a^	86^a^
Obstetrician-led care	15 (8.0)	21	14
Referred during pregnancy	36 (19.3)	37	35
Care during labor			
Midwife-led care	99 (52.9)	42^b^	51^b^
Obstetrician-led care	51 (27.3)	58	49
Referred during labor	37 (19.8)	16	22

PRN data are national data. Regional data are Perined-insight LVR2 data

In the regional and national comparison groups, women with a pre-labor caesarian section and who had a child with a major congenital abnormality could not be excluded

^a^including women who were referred during pregnancy

^b^including women who were referred during labor

Of the 790 women who were asked to participate, 325 (41%) gave written informed consent and were invited to complete the online questionnaire. One hundred ninety five of the 325 women who gave informed consent (60%), completed the online questionnaire. Eight women were excluded of whom six had a primary caesarean section and two gave birth to a child with a congenital abnormality.

Of all participants 41.7% were primiparous and 87.2% had a spontaneous vaginal birth. 36 (19.3%) women were referred during pregnancy and 37 (19.8%) women were referred during labor to obstetrician-led care. Of the 15 women in obstetrician led care from the onset of pregnancy 12 women had a spontaneous vaginal birth, 1 woman had an assisted vaginal birth and 2 women had an emergency caesarian section (data not shown).

Compared to all women who gave birth in 2014 in the whole region of Leiden our study population had a higher percentage of spontaneous vaginal births and home births (Table 1).

In Table 2 the mean scores for three subscales of the NCQ are presented. The Cronbach’s Alpha values for women who were not referred during pregnancy successively for the three subscales were 0.81, 0.75 and 0.84 and for women who were referred during pregnancy 0.84, 0.74 and 0.83 respectively. Women who were in midwife-led care and who were not referred during their pregnancy had statistically significantly higher mean scores for continuity of care compared to those in obstetrician-led care in all subscales. Regression analyses adjusted for parity showed the same results.

**Table 2:** Experienced continuity of care by women measured with subscale scores of the Nijmegen Continuity Questionnaire

	Not referred during pregnancy (*n* = 151)		Referred during pregnancy (midwife-led care at onset of pregnancy and obstetrician-led care at onset of labor) (*n* = 36)	
Total of the Subscale	Midwife-led care at onset of pregnancy and labor. Score primary care midwife ^¥^	Obstetrician-led care at onset of pregnancy and labor. Score hospital staff	Score primary care midwife	Score hospital staff
	mean (*n* = 136) ^¥^	mean (*n* = 15)	mean (*n* = 36)	mean (*n* = 36)
Subscale 1: personal continuity/ care provider knows me				
1. I know this care provider very well	3.71	3.20	3.57	2.58
2. This care provider knows my medical history very well	4.10	3.93	3.91	3.34
3. This care provider always remembers what he/she did during my last visit(s)	4.08	3.80	3.96	3.27
4. This care provider knows my family circumstances very well	3.77	3.13	3.77	2.60
5. This care provider knows very well what I do in my day-to-day life	3.53	2.80	3.46	2.40
Total subscale score	3.84	**3.37 (*p*** **= 0.02)**	3.73 (*p* = 0.44)	**2.83 (*p*** **= <0.001)**
missing	6	0	1	6
Subscale 2: Personal continuity/ care provider shows commitment				
This care provider contacts me when necessary without me having to ask him/her to do so	3.84	2.60	3.89	2.22
2. This care provider knows very well what I think is important when it comes to my care	3.93	3.13	3.83	2.39
3. This care provider maintains enough contact with me when I am seen by another care provider	n.a.	n.a.	4.03	2.70
Total subscale	n.a.	n.a.	3.94	2.42
missing			2	4
Subscale 3: Team continuity				
1. These care providers pass on information to each other very well	4.27	3.67	4.11	3.25
2. These care providers work together very well	4.36	3.73	4.09	3.53
3. The care given by these care providers is well-connected	4.33	3.67	4.17	3.59
4. These care providers always know very well what the other care providers have done	4.23	3.60	4.09	3.44
Total subscale score	4.29	**3.67 (*p*** **= 0.002)**	4.11 (*p* = 0.20)	**3.43 (*p*** **= <0.001)**
missing	4	0	1	6
Subscale 3b Cross-boundary continuity	n.a.	n.a.	3.38	
missing			4	

Experienced care for women who received midwife-led care at onset of labor compared to a. obstetrician-led care, at onset of labor and b. care provided by primary care midwives and hospital staff for women who were referred during pregnancy. (Subscale scores of the NCQ: *n* = 187 women)

Mean score (1 = strongly disagree, 2 = disagree, 3 = neutral, 4 = agree, 5 = strongly agree)

n.a. No score because one item is not applicable (see additional file)

^¥^Reference category

Statistically significant results *p* < 0.05 are in bold

Women who were referred during their pregnancy had similar mean scores for continuity of care by the midwife compared to women in midwife-led care who were not referred. Women who were referred during pregnancy had lower mean scores for continuity of care by hospital staff compared to women in midwife-led care who were not referred. However, not all differences were statistically significant.

The mean score for cross-boundary continuity of care of women referred during labor was higher compared to women referred during pregnancy (3.62 versus 3.38; difference not tested). (Additional file 1: Table S1). Regression analysis adjusted for parity showed similar result.

Table 3 shows the score of the PCQ with a Cronbach’s Alpha of 0.87 and the score of the CPS with a Cronbach’s Alpha of 0.75.

**Table 3:** Experienced quality of care during labor measured with the Pregnancy and Childbirth Questionnaire and perception of labor measured with the Childbirth Perception Scale

	Not referred during pregnancy (*n* = 151)		Referred during pregnancy (*n* = 36)
Total scale score	Midwife-led care at onset of labor mean (*n* = 136) ^¥^	Obstetrician-led care at onset of labor mean (*n* = 15)	
a. PCQ	4.23	4.13 (*p* = 0.55)	4.08 (*p* = 0.22)
missing	3	3	1
b. CPS Total subscale score	2.30	**2.02 (*p*** **= 0.04)**	**1.95 (*p*** **= <0.001)**
missing	3	0	0

Experienced quality of care during labor and perception of labor for women who received midwife-led care at onset of labor compared to a. obstetrician-led care, at onset of labor and b. women who were referred during pregnancy (scores of the PCQ and CPS; *n* = 187 women)

a Mean score (after recoding) (1 = strongly disagree, 2 = disagree, 3 = neutral, 4 = agree, 5 = strongly agree)

b Mean score (after recoding) (0 = strongly disagree, 1 = disagree, 2 = agree, 3 = strongly agree)

*P < 0.05

^¥^Reference category

Statistically significant results *p* < 0.05 are in bold

The results were similar for both scales: the score for women who were in midwife-led care was highest and women who were referred during pregnancy scored lowest but only the differences in CPS scores were statistically significant. (Additional file 1: Table S2 and S3 show this in more detail).

Table 4 shows the association between experienced continuity of care and experienced quality of care during labor.

**Table 4:** Correlation between experienced continuity of care measured with the Nijmegen Continuity Questionnaire and experienced quality of care during labor measured with the Pregnancy and Childbirth Questionnaire

	NCQ Subscale 1: personal continuity/ care provider knows me		NCQ Subscale 2: Personal continuity/care provider shows commitment		NCQ Subscale 3: Team/cross-boundary continuity	
		*P* value		*P* value		*P* value
Not referred						
Midwife-led care (*n* = 136)	**0.40**	**<0.001**	**0.41**	**<0.001**	**0.54**	**<0.001**
Obstetrician-led care (*n* = 15)	0.43	0.11	0.42	0.12	0.44	0.10
Referred						
Score hospital staff (*n* = 36)	**0.47**	**0.009**	0.29	0.10	**0.41**	**0.025**

Spearman correlation between experienced continuity of care (NCQ) and experienced quality of care during labor (PCQ) for women who received midwife-led care at onset of labor, obstetrician-led care at onset of labor and care provided by hospital staff for women who were referred during pregnancy

Statistically significant results *p* < 0.05 are in bold

For personal continuity (“the care provider knows me”), a moderate correlation with quality of care was found for women in midwife-led care (*r* = 0.40, *p* < 0.001). For women who were referred during pregnancy a moderate correlation was found for the scores for hospital staff (*r* = 0.47, *p* = 0.009).

The second subscale on personal continuity (“the care provider shows commitment”) showed a moderate correlation with quality of care for women in midwife-led care (*r* = 0.41, *p* = 0.025).

For team continuity a strong correlation with quality of care was found for women in midwife-led care (*r* = 0.54, *p* < 0.001). A moderate correlation with quality of care during labor was found in the referred group for the scores for hospital staff (*r* = 0.41, *p* = 0.025).

Regression analyses adjusted for parity showed the same results.

Table 5 shows the correlations between experienced continuity of care and women’s perception of labor.

**Table 5:** Correlation between experienced continuity of care measured with the Nijmegen Continuity Questionnaire and women’s perception of labor measured with the Childbirth Perception Scale

	NCQ Subscale 1: personal continuity/care provider knows me		NCQ Subscale 2: Personal continuity/care provider shows commitment		NCQ Subscale 3: Team/cross-boundary continuity	
		*P* value		*P* value		*P* value
Not referred during pregnancy						
Midwife-led care (*n* = 136)	**−0.21**	**0.016**	**−0.31**	**0.002**	−0.12	0.17
Obstetrician-led care (*n* = 15)	−0.17	0.54	0.14	0.62	0.08	0.79
Referred during pregnancy						
Score hospital staff (*n* = 36)	−0.19	0.31	0.06	0.75	−0.16	0.39

Spearman correlation between experienced continuity of care (NCQ) and women’s perception of labor (CPS) for women who received midwife-led care at onset of labor, obstetrician-led care at onset of labor and care provided by hospital staff for women who were referred during pregnancy

Statistically significant results *p* < 0.05 are in bold

For subscale 1 regarding personal continuity (“the care provider knows me”), a weak negative correlation with perception of labor was found for women in midwife-led care (*r* = −0.21, *p* = 0.016).

For the second scale of personal continuity (“the care provider shows commitment”) a moderately negative correlation with perception of labor was found for midwife-led care (*r* = −0.31, *p* = 0.002). For team continuity no significant correlations were found.

## Discussion

The experienced personal and team continuity of care during pregnancy was significantly higher for women in midwife-led care compared to those in obstetrician-led care. Experienced continuity of care during pregnancy was moderately correlated with experienced quality of care although not significantly so in all subgroups A weak negative correlation was found between experienced personal continuity of care by the midwife and perception of labor.

The findings that the level of experienced personal continuity of care was higher among women in midwife-led versus obstetrician-led care is in line with the literature showing that continuity of care has been identified as a core component of a midwife-led care model [4]. Logically, women experience less cross boundary continuity if they are referred from primary to secondary care, as they will receive care from a new team of caregivers. However, our study did not show that women who were referred during pregnancy experienced less personal continuity of care from all professionals compared to women solely under obstetrician-led care. An explanation for this could be that in our study women in the hospital are attended by different caregivers (e.g. clinical midwife, nurse, resident and obstetrician).

The cross boundary continuity is higher if women are referred during labor compared to if they are referred during pregnancy. This may be related to the fact that in case of referral for certain indications such as failure to progress during the second stage and suspected fetal distress during labor, the midwife usually remains present during the entire labor.

It will be important to evaluate the effect of integrating midwife-led and obstetrician-led care on the different aspects of continuity of care.

A weak or no correlation was found between the experienced continuity of care during pregnancy and the perception of labor. These findings suggest that other aspects than continuity of care are important for women’s perception of labor. This is in accordance with the literature [25], which shows that the patient perspective in maternity care is complex, and multidimensional. Unfortunately, scales measuring childbirth experience fail to capture this complexity [25].

Literature shows that continuity of care is important to women. Although Posthumus et al. [26] describe that in a model of shared care, continuity of care could be improved, we should also be aware that integration of midwife-led and obstetrician-led care could be at the expense of continuity of care for women. As more caregivers will be involved in an integrated care system this could lead to less experienced continuity of care for women. Working in small teams of caregivers, especially in hospitals could be of great benefit. This will be a great challenge, especially in hospitals in the Netherlands, as teams usually include a large number of caregivers. Small teams in which women are seen by a minimum number of caregivers, could result in more continuity of care as well [20, 27].

A strength of this study is that a comparison could be made between the experienced continuity of care, the experienced quality of care and perception of labor because the same women completed all questionnaires.

Our study has some limitations as well: the response rate was low with nearly 25% of the eligible women taking part and the total number of women in obstetrician-led care was small. The percentage of women in midwife-led care in our sample was high, also compared to national data. Possibly midwives were more alert (or prone) to include women whom they had taken care of during their pregnancy. Also, this response bias could have impacted the results of this survey as literature shows a positive correlation between patient satisfaction and response rate [28]. If the response rate had been higher, the scores for experienced continuity, satisfaction and birth experience might therefore have been different. In addition, patients do not easily express dissatisfaction [13], which could have led to an overrepresentation of positive experiences in our results, in particular about primary care midwives. Furthermore, the NCQ [21]was developed for patients with a chronic disease in general practice whereas we used it to measure experienced continuity of care during pregnancy. As we are not certain whether our population scores the same on the NCQ, we recommend validation of the NCQ for women in perinatal care in future research. Although it is possible that part of the variation can be explained by clustering of respondents in midwifery practices, multi-level analyses were not performed because the number of respondents per practice was not sufficient for meaningful analyses. For the same reason, we did not adjust for complications because of the small size of groups. This could have resulted in higher scores for obstetrician-led care.

The questionnaires we used varied from general questions (NCQ) regarding pregnancy and labor to specific questions with regards to labor (CPS and PCQ). Therefore, they are not fully comparable.

Finally, the time between giving birth and completing the questionnaire varied from one to 6 months. As women’s perceptions of their experience of birth changes over time, this could have influenced our results. Women who filled in the questionnaire soon after birth might have been more positive about their care compared to those who filled it in after several months [29].

## Conclusion

This study suggests that experienced continuity of care depends on the care context because scores were higher for midwife-led care compared to obstetrician-led care. It will be a challenge to maintain the high level of experienced continuity of care in an integrated maternity care system. Experienced continuity of care and experienced quality of care during labor were only associated for women who were not referred during pregnancy. Experienced continuity of care seems to be a distinctive concept that should not be confused with experienced quality of care or perception of labor and should be considered as a complementary aspect of quality of care.

## Additional file

Additional file 1: Table S1. Cross-boundry continuity. The mean score for cross-boundary continuity of care for women referred during pregnancy and women referred during labor. Table S2 Item means and total scale score of the Pregnancy and Childbirth Questionnaire. Table S3 Item means and total subscale score of the Childbirth Perception Scale. (DOCX 18 kb)

## Abbreviations

CPS: Childbirth Perception Scale
NCQ: Nijmegen Continuity Questionnaire
PCQ: Pregnancy and Childbirth Questionnaire
