# Supplementary material for: Continuity of care is an important and distinct aspect of childbirth experience: findings of a survey evaluating experienced continuity of care, experienced quality of care and women’s perception of labor
Source: BMC Pregnancy Childbirth. 2018 Jan 8;18:13. doi: 10.1186/s12884-017-1615-y (PMC5759271; doi:10.1186/s12884-017-1615-y)
Supplement: Additional file 1: Table S1. — Cross-boundry continuity. The mean score for cross-boundary continuity of care for women referred during pregnancy and women referred during labor. Table S2 Item means and total scale score of the Pregnancy and Childbirth Questionnaire. Table S3 Item means and total subscale score of the Childbirth Perception Scale. (DOCX 18 kb) [file 12884_2017_1615_MOESM1_ESM.docx]

**Additional file**

**Additional file Table 1.** Cross-boundary continuity

| **Subscale 3: Cross-boundary continuity** | **Referred during pregnancy (n=36)** | **Referred during labor (n=37)** |
| --- | --- | --- |
| 1. These care providers pass on information to each other very well | 3.41 | 3.68 |
| 2. These care providers work together very well | 3.36 | 3.74 |
| 3. The care given by these care providers is well-connected | 3.44 | 3.67 |
| 4. These care providers always know very well what the other care providers have done | 3.31 | 3.39 |
| Total subscale score  missing | 3.38  4 | 3.62  4 |

Mean score (1=strongly disagree, 2=disagree, 3=neutral, 4=agree, 5=strongly agree)

**Additional file Table 2.** Item means and total scale score of the Pregnancy and Childbirth Questionnaire.

|  | **Not referred during pregnancy**  **(n=151)** | | **Referred during pregnancy**  **(n=36)** |
| --- | --- | --- | --- |
|  | Midwife-led care at onset of pregnancy and labor. Score primary care midwife  mean  (n=136) | Obstetrician-led care at onset of pregnancy and labor. Score hospital staff  mean  (n=15) | mean  (n=36) |
| Keeping informed on the progress of birth | 4.32 | 4.33 | 4.06 |
| Paid attention to partner during delivery | 4.27 | 3.87 | 4.17 |
| Being aware of preferences and wishes | 4.14 | 3.87 | 3.94 |
| Good communication with professionals during delivery | 4.41 | 4.33 | 4.20 |
| Communication between professionals | 4.29 | 4.27 | 4.14 |
| Clear who is in charge of care during delivery | 4.34 | 4.40 | 4.09 |
| Involved in decision making regarding analgesia | 3.86 | 3.87 | 4.00 |
| Total scale score  missing | 4.23  3 | 4.13  3 | 4.08  1 |

Mean score (1=strongly disagree, 2=disagree, 3=neutral, 4=agree, 5=strongly agree)

**Additional file Table 3.** Item means and total subscale score of the Childbirth Perception Scale.

|  | **Not referred during pregnancy (n=151)** | | **Referred during pregnancy**  **(n=36)** |
| --- | --- | --- | --- |
|  | Midwife-led care at onset of pregnancy and labor. Score primary care midwife  mean  (n=136) | Obstetrician-led care at onset of pregnancy and labor. Score hospital staff  mean  (n=15) | mean  (n=36) |
| My labor was worse than I expected* | 0.57 | 1.13 | 1.22 |
| I felt safe during my labor | 2.19 | 2.0 | 2.03 |
| When I was in labor I did many things wrong* | 0.38 | 0.53 | 0.64 |
| When I was in labor I doubted whether I would be able to do it* | 0.79 | 1.27 | 1.11 |
| I panicked during my labor* | 0.65 | 0.67 | 0.75 |
| I was able to relax during my labor | 1.98 | 1.73 | 1.39 |
|  | | | |
| Total subscale score  missing | 2.30  3 | 2.02  0 | 1.95  0 |

Mean score (0=strongly disagree, 1=disagree, 2=agree, 3=strongly agree)

* for analysis the scores for negative stated CPS questions were reversed.
